# A genome wide association study to dissect the genetic architecture of agronomic traits in Andean lupin (*Lupinus mutabilis*)

**DOI:** 10.3389/fpls.2022.1099293

**Published:** 2023-01-04

**Authors:** Agata Gulisano, Antonio Lippolis, Eibertus N. van Loo, Maria-João Paulo, Luisa M. Trindade

**Affiliations:** ^1^ Wageningen University and Research Plant Breeding, Wageningen University, Wageningen, Netherlands; ^2^ Wageningen University and Research Biometris, Wageningen Research, Wageningen, Netherlands

**Keywords:** *Lupinus mutabilis*, molecular markers, SNP, flowering time, association mapping, plant architecture

## Abstract

Establishing *Lupinus mutabilis* as a protein and oil crop requires improved varieties adapted to EU climates. The genetic regulation of strategic breeding traits, including plant architecture, growing cycle length and yield, is unknown. This study aimed to identify associations between 16 669 single nucleotide polymorphisms (SNPs) and 9 agronomic traits on a panel of 223 *L. mutabilis* accessions, grown in four environments, by applying a genome wide association study (GWAS). Seven environment-specific QTLs linked to vegetative yield, plant height, pods number and flowering time, were identified as major effect QTLs, being able to capture 6 to 20% of the phenotypic variation observed in these traits. Furthermore, two QTLs across environments were identified for flowering time on chromosome 8. The genes *FAF*, *GAMYB* and *LNK*, regulating major pathways involved in flowering and growth habit, as well as *GA30X1*, *BIM1*, *Dr1*, *HDA15*, *HAT3*, interacting with these pathways in response to hormonal and environmental cues, were prosed as candidate genes. These results are pivotal to accelerate the development of *L. mutabilis* varieties adapted to European cropping conditions by using marker-assisted selection (MAS), as well as to provide a framework for further functional studies on plant development and phenology in this species.

## 1 Introduction

*Lupinus mutabilis*, also known as “Andean lupin”, is an endemic legume of the Andean region of South America. Firstly domesticated in the north of Peru (Atchison et al., 2016), *L. mutabilis* has been traditionally cultivated in Ecuador, Peru and Bolivia for soil enrichment and as a food crop (Gross and von Baer, 1981). Similarly to other Andean grains, in the last 500 years its cultivation has been marginalized and neglected due to the introduction of western pulses characterized by higher productivity. The scarce dissemination of its nutraceutical properties, and the presence of alkaloids providing a bitter taste to the seeds, have also partly contributed to this negligence (Chirinos-Arias et al., 2015). Only in the past decades, the demand for alternative plant protein sources and the need to maximize agricultural land, by making use of marginal lands, has sparked renewed interest in this crop, not only in South America but also in Europe. *Lupinus mutabilis* seeds are characterized by a high content of protein and oil (44% dm and 18% dm, on average respectively), which exceeds that of any other lupin species and is comparable to soybean. On top of that, *L. mutabilis* is adapted to low input farming in temperate climates and can effectively contribute to the improvement of poor soils by fixing nitrogen and mobilizing soil phosphates. In Europe and other temperate climatic regions, the combination of these features makes *L. mutabilis* a potentially superior alternative to current plant-based sources of protein and oil. Despite this, some challenges need to be addressed to expand its cultivation on large scale, as *L. mutabilis* remains to date an under studied crop. Pivotal to achieving this aim are breeding programs focused on guaranteeing economic viability and consumer acceptance of the crop.

In the past decades, numerous studies have investigated the nutritional profile and potential applications of *L. mutabilis* seeds in a wide range of end-use products, from protein, oil and food additives to cosmetics, medicines and bio-pesticides. In contrast, few studies have addressed the agronomic aspect of its cultivation. Research on the adaptation of *L. mutabilis* to European soil and climate conditions started only 30 years ago, when the first European project focusing on 16 selected lines was initiated. More recently, a second European project (LIBBIO) has expanded this investigation to 225 *L. mutabilis* accessions, by evaluating a wide panel of Andean germplasm both as a winter crop in Mediterranean conditions and as a summer crop in North-Central European conditions. Both projects have pointed out the need of breeding for a better plant architecture, early maturity and yield stability (Caligari et al., 2000; Gulisano et al., 2022a). These traits are highly interconnected. Plant architecture has been identified as the main factor limiting yield in European environments (Caligari et al., 2000). This is to a great extent due to the indeterminate growth habit characterizing *L. mutabilis*, which leads to an overlap of vegetative and reproductive phases, hindering uniform maturation and delaying reproductive growth. The ability to prolong indefinitely vegetative growth after the onset of flowering, results in flowers and pods abortion in temperate climatic conditions during the long maturation period (Galek et al., 2007). Conversely, in Mediterranean environments, dry conditions at the end of the cropping season can drastically affect biomass yield and pods set, decreasing considerably seed yield (Hardy et al., 1997). Early flowering genotypes can contribute to shorten the growing season and to escape terminal drought and heat stress (Gulisano et al., 2022a), but only the combination of determinacy and earliness can ultimately lead to higher and more stable seed yield.

Similar challenges have characterized the domestication of other legumes, including “old world” lupin species. Indeterminate growth habit is typical of many wild relatives of grain legumes (e.g. pea, soybean, common bean) and its switch to determinate growth can be considered as one of the most important traits of their domestication (Krylova et al., 2020). The identification of genes responsible for growth habit has been pivotal for the development of determinate varieties, and has highlighted the interconnection of growth habit with stem length, flowering duration, yield, resistance to lodging and suitability to mechanized cultivation (Krylova et al., 2020). Mutation of *TERMINAL FLOWER 1* (*TFL1*)-like gene controlling transition to flowering has led to determinate growth habit in many legumes, including pea (Foucher et al., 2003), faba bean (Avila et al., 2006), common bean (Kwak et al., 2008) and soybean (Tian et al., 2010). *TFL1* belongs to the small gene family *CENTRORADIALIS/TERMINAL FLOWER1/SELF–PRUNING (CЕTS)*, controlling the developmental transition from indeterminate to determinate growth habit (Wickland and Hanzawa, 2015). Besides regulating flowering, genes of the *CETS* family are also involved in other processes, including stomatal opening or gibberellic and abscisic acid signaling pathways (Xi et al., 2010; Ando et al., 2013). Within the Lupin genus, determinate cultivars have been obtained in *L. albus, L. luteus*, and *L. angustifolius* through selection of spontaneous or induced mutants. The first determinate types in *L. mutabilis* have been obtained through induced mutation in Poland, distinguished by medium-tall stems without lateral branches, resistance to lodgings and early generative growth (Galek et al., 2007). Nevertheless, the molecular mechanisms underlying plant-architecture and other yield related traits remains still unknown in *L. mutabilis*, due to the lack of genetic and molecular studies on this crop.

Given the importance of these agronomic traits, a large number of genomic regions (Quantitative Trait Loci, QTLs) associated to plant-architecture related traits, flowering time and seed yield has been identified in the past decades for many grain legumes (Dargahi et al., 2014; Yao et al., 2015; Ávila et al., 2017; Klein et al., 2020). These studies have contributed to significantly increase knowledge on the genetic basis of these traits and to accelerate breeding of these crops. However, the majority of them has focused on identifying QTLs in biparental populations, hence limiting genetic variation and mapping resolution (Gupta et al., 2005). With the current development of sequencing and genotyping technologies at affordable cost, genome wide association studies (GWAS) have rapidly become a more common and powerful tool to investigate natural variation and to identify genomic regions underlying important agronomic traits. Moreover, GWAS approach allows to exploit higher phenotypic diversity than biparental mapping populations derived from targeted crosses, as well as a direct application of the results from research to breeding.

In this study, we evaluated a panel of 223 diverse accessions of *L. mutabilis* in the native Andean region, and over two cropping conditions in Europe, in order to identify Single Nucleotide polymorphisms (SNPs) underlying the variation in plant-architecture, flowering and yield related traits in this species. A GWAS approach was used to capture the natural diversity present in the panel, with the objective to develop genetic markers and highlight genomic regions (QTLs) harboring causal candidate genes, which are critical for assisting and speeding up the breeding of *L. mutabilis.*

## 2 Material and methods

### 2.1 Plant materials and field trials

The GWAS panel used in this study comprised 223 *L. mutabilis* accessions, 201 provided by the Instituto Nacional de Investigaciones Agropecuarias of Quito (INIAP, Ecuador), comprising landraces, varieties and wild material collected across the Andean region, and 22 *L. mutabilis* lines developed in European breeding programs. The panel was evaluated in a total of four field trials in Ecuador and Europe during the growing seasons 2019 and 2020. The locations were chosen to represent an example of cultivation in the native environment as well as in Mediterranean (winter cycle) and North-Central European (summer cycle) climates and photoperiod regimes. The trial in Ecuador (EC, Cotopaxi 0° 55´ 35´´ S, 78° 40´ 07.4´´W) was sown in December 2019 and harvested in June 2020. The field layout was an alpha-lattice design with 3 replicates. Plants were arranged in plots with 5 rows (80 cm between rows), containing 40 plants spaced 20 cm. In Europe, the field trials were set up during winter (Nov 2019 – May 2020) for the Portuguese site (PT, Lisbon 38° 42´ 33.5´´ N, 9° 11´ 0.5´´W), and during two consecutive growing seasons from April to October in two locations in The Netherlands (NL-Sc 2019, Scheemda 53° 09´ 60´´ N, 6° 57´ 59.9´´E; NL-Wi 2020, Winschoten 53° 10´ 11.´´ N, 7° 2´ 56.09´´E). A randomized complete block design (RCBD) with three replicates was adopted for the three EU trials. Out of 20 plants present in each plot, at a distance of 30x30 cm, phenotyping was conducted on the 6 central plants. In all the locations, plants were cultivated under rain-fed conditions and without the aid of any fertilization, following local cultivation practices.

### 2.2 Phenotyping of the GWAS panel

The phenotypic evaluation of the *L. mutabilis* GWAS panel across the four environments included the scoring of quantitative traits related to plant morphology, phenology and agronomic performance (Gulisano et al., 2022a). The phenotypes of interest were: flowering time, plant height, number of branching orders, vegetative yield, number of pods and seeds produced on the main stem, total number of pods and seeds produced on the overall plants, and 100 seeds weight. The traits were scored as described in our previous study (Gulisano et al., 2022a). Briefly, flowering time was scored as number of days from sowing until 50% of the plants in a plot had started flowering. Due to unforeseen circumstances, longer intervals in scoring of flowering in NL-Sc resulted in suboptimal phenotyping, as also shown by the low heritability estimate for flowering in this trial (H^2^ = 0.24; Gulisano et al., 2022a), hence these data were excluded from this study. Instead, in Ecuador, due to the impossibility of collecting data during Covid restrictions, scoring of flowering time and vegetative yield was not possible. Phenotyping was conducted on the six central plant of the plots (five in Ecuador). At harvest, height of the main stem (cm) and number of branching orders were scored (0 = main stem only, 1 = main stem and first branching order, etc.). Number of pods on the main stem (Pods MS) and total number of pods (Pods T) was also recorded. After harvesting, seeds were air-dried and counted separately on the main stem (Seeds MS) and on the total plant (Seeds T). Vegetative yield (dw, g/plant) was estimated as the difference between the total amount of biomass harvested (dw) and the seed yield per plant.

### 2.3 Statistical analysis

Phenotypic data were analyzed using the SpATS mixed model approach, implemented in *statgenSTA* R package (v1.0.8), to correct for spatial gradients in the field by adopting a 2-dimensional smoothing with P-splines (Rodriguez-Alvarez et al., 2018). The analysis was conducted in a two-stages approach, where the final adjusted mean across trial was calculated after performing a single trial analysis. The best linear unbiased estimations (BLUEs) of genotypic means were obtained from the models and then used for the rest of the analyses. In addition, a random effects model was fitted using *lme4* R package to calculate variance components of genotype (G), environment (E) and genotype by environment interaction (GEI) and estimates of broad sense heritability across trials. Broad sense heritability was calculated across the three environments as in (Renaud et al., 2014):


$$H^{2}=V_{G}/\left(V_{G}+\frac{V_{GEI}}{nE}+\frac{V_{\epsilon }}{nE*nBlock}\right)$$

where *VG*, *VGE*I, *V_ϵ_* represent respectively the estimated genetic, GEI and error variance components, while *nE* represents the number of environments and *nBlock* the number of blocks in each environment. Pearson’s correlations between BLUEs genotypic values in each trial were estimated and plotted using the R package *corrplot* (v0.92).

### 2.4 Genotyping and SNP development

Reduced representation sequencing and single nucleotide polymorphism (SNP) typing was performed as previously described in Gulisano et al. (2022a). Briefly, genomic DNA was isolated from young grinded *L. mutabilis* leaves (∼20–400 mg, freeze dried material) using acetyl trimethyl ammonium bromide (CTAB) method (Doyle and Doyle, 1987) as described in Petit et al. (2020). To cover some degree of genetic heterogeneity expected in the accessions, DNA was extracted from a pool of 10 individuals/accession. Restriction site-associated DNA sequencing (RAD-seq) was used to identify SNPs distributed over the genome, by digesting 1 ug of high-quality genomic DNA (at a concentration ≥ 25 ng/μl) using the restriction enzyme EcoRI. RAD library preparation and sequencing were performed by Beijing Genomics Institute (BGI, Hong Kong). The Burrows–Wheeler Alignment Tool based on BWA- MEM algorithm were used to map the clean sequence reads to the *L. angustifolius* ‘Tanjil’ (LupAngTanjil_v1.0 refSeq GCF_001865875.1) genome reference (Hane et al., 2017). The average mapping rate was 76.9%, and the properly paired average 63.7%. BCFtools (v1.9) was used to call SNPs in each sample based on genotype likelihoods. At each locus, SNPs were called as percentage of the reference allele present on the total number of reads generated. After SNPs quality control and removal of SNPs not assigned to any chromosome, a total set of 16,781 biallelic SNPs was selected for the genetic analysis.

### 2.5 Genome-wide association studies

Single trait GWAS analysis was conducted separately for each environment, using 16,669 polymorphic markers after filtration to remove SNPs with minor allele frequencies (MAF)< 0.02. The GWAS model was based on a linear mixed model for association mapping as implemented in StatgenGWAS package v1.0.5 (van Rossum et al., 2020). Single trait GWAS in statgenGWAS follows the approach of Kang et al. (2008), by performing a two steps procedure. Firstly, an ‘empty’ model without any SNP effect is fitted in order to obtain REML-estimates of the genetic and residual variance components, computed using the Efficient Mixed Model Association (EMMA) algorithm (Kang et al., 2008). Secondly, the single SNP-effect of interest is tested by using generalized least-squares (GLS) and F-test, obtaining the effect-size and P-values for all SNPs. Population structure and individuals’ relatedness were taken into account by fitting a Van Raden kinship matrix and adding origin of the accessions as corrections. The adequateness of genetic relatedness correction was assessed by evaluating genomic inflation factors. The Bonferroni correction was used to correct for multiple testing, thus obtaining a threshold of 5.52 for –log_10_ (p) that was used to state statistically significant SNPs. Manhattan plots were visualized using *qqman* package in R (Turner, 2018). Linkage disequilibrium for this panel was already estimated in Gulisano et al. (2022a), following the approach of (Vos et al., 2017). LD was estimated to decay around 80 kbp of distance.

### 2.6 Candidate genes identification

The size of the genomic regions investigated to identify putative candidate genes, controlling the traits under study, was defined by the extend of the average Linkage Disequilibrium across the genome. Starting from the position of the detected significant SNPs, candidate genes were proposed whether harbored in a maximum physical distance, upstream and downstream, of 80 kbp. All candidate genes were selected based on the information contained in the NCBI *Lupinus angustifolius* Annotation Release 100 for the genome assembly GCF_001865875.1 of LupAngTanjil_v1.0 (https://ftp.ncbi.nlm.nih.gov/genomes/all/annotation_releases/3871/100/). Special attention was given to genes with predicted functions related to the flowering time and regulation of plant growth and development, based on gene description and relevant research paper.

## 3 Results

### 3.1 Phenotypic analysis

**Figure 1:**
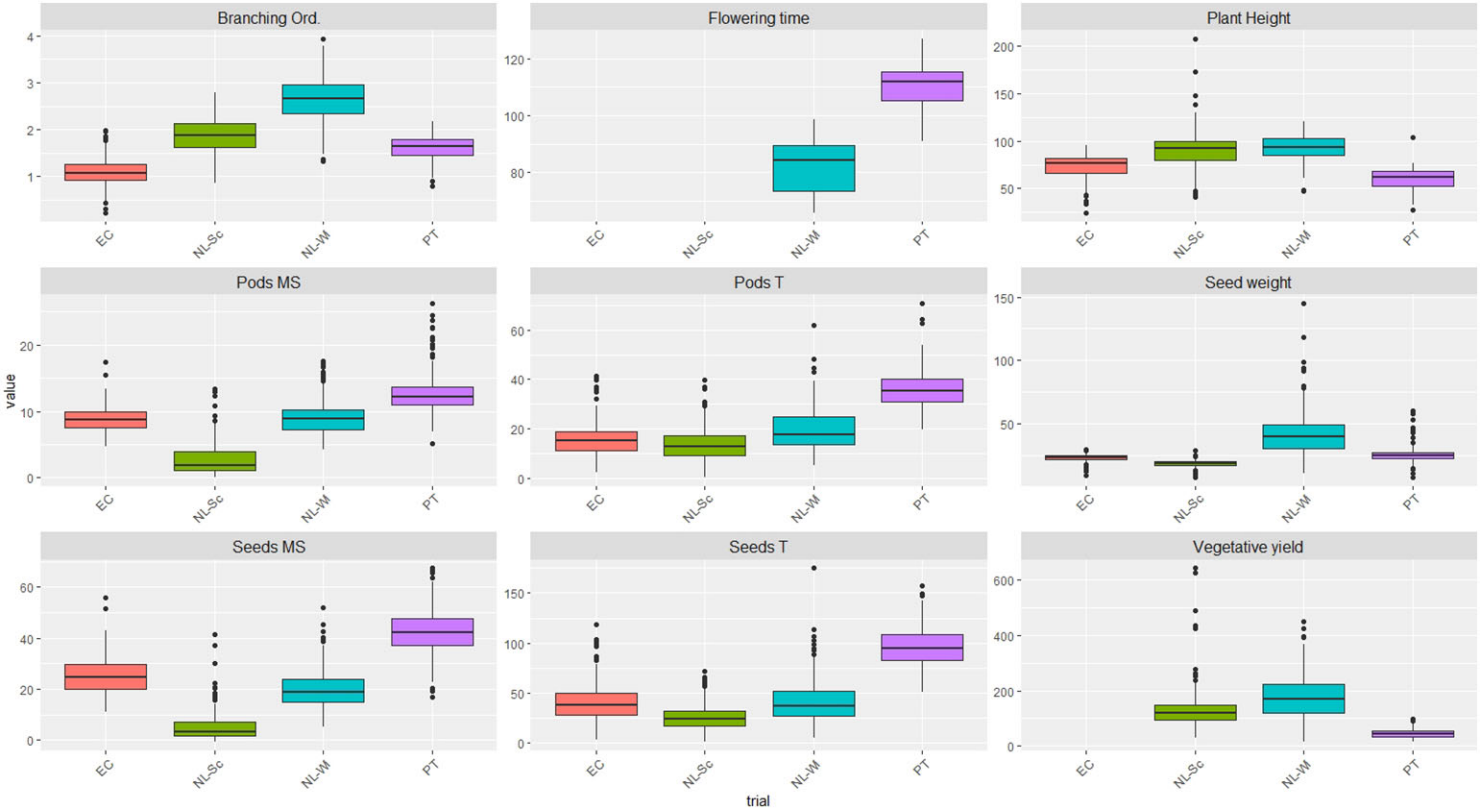
Boxplots showing the variation of the plant architecture and yield related traits (BLUES data) under study in a collection of 223 *L. mutabilis* accessions. The upper and lower end of the boxes indicate respectively the 75th and 25th percentiles, while the line in the middle represents the 50th percentile (median). The whiskers represent the highest and lowest values, while circles represent outliers outside the 5th-95th percentile interval.

Nine traits relevant for breeding of *L. mutabilis* accessions adapted to different cropping conditions in Europe and Ecuador were investigated using a panel of 223 accessions. Extensive phenotypic variations were observed for all traits, namely flowering time, plant height, number of branching orders, vegetative yield, number of pods and seeds on the main stem, total number of pods and seeds and seed weight (depicted in **Figure 1**). Remarkable variation in flowering time was observed between the two European cropping conditions. In particular, flowering occurred at an earlier time in the Netherland than Portugal, with an average of 81 and 110 days for NL-Wi and PT respectively. Relevant phenotypic diversity in time to flowering was observed within the panel, as highlighted by coefficients of variation (CV %) of 6% in Portugal and 8% in the Netherlands 2020. The overall plant height across different locations ranged from 24 cm to and 248 cm, indicating that *L. mutabilis* small genotypes can be 10 times smaller than tall genotypes as combination of environmental and genetic effects. As general trend, plants were shorter on average in PT (60 cm) and EC (73 cm) compared to NL-Sc (90 cm) and NL-Wi (92 cm) and characterized on average by a first branching order only. Contrarily, in NL-Sc and NL-Wi the main stem reached an average height of 90 and 92 cm respectively, with two/three branching orders produced. Plant height had high phenotypic variability within the 223 accessions, as pointed by the CV values of 17%, 18%, 20%, 13% in Ecuador, Portugal, NL-Sc and NL-Wi respectively. Vegetative yield reflected the development of *L. mutabilis* in different environments. In Portugal, the average vegetative yield recorded (45.8 g/plant) was between three and four times lower than the average yield of NL-Sc (133.6 g/plant) and NL-Wi (174.9 g/plant). Concerning grain yield components, the average number of pods and seeds produced by the total plant, as well as by the main stem only, was higher in winter-Mediterranean conditions, followed by the native environment (EC) and summer-North European environments. The number of seeds harvested per plant was on average 42 in EC (CV 47%), 95 in PT (CV 19%), 26 in NL-Sc (CV 49%) and 42 (CV 53%) in NL-Wi. The number of pods and seeds produced on the main stem were respectively 9 (CV 20%) and 25 (CV 28%) in EC, 13 (CV 23%) and 42 (CV 21%) in PT, 3 (CV 89%) and 14 (CV 47%) in NL-Sc and 9 (CV 27%) and 20 (CV 37%) in NL-Wi. Conversely, the yield of biomass agricultural residues was lowest in PT (45.8 g/plant) and highest in NL-Wi (174.9 g/plant). An exhaustive analysis of the phenotypic traits under study was conducted in a previous study of diversity and agronomic adaptation of this collection. There, the mean values, range of variation and Pearson’s correlation between plant architecture and yield-related traits are reported (Gulisano et al., 2022a).

### 3.2 Heritability estimates and identification of relevant breeding traits

In the four separate locations, estimates of heritability were high for flowering time (0.87-0.93) and plant height (0.5-0.8). Moderate to high heritability was also estimated for number of pods on the main stem (0.4-0.8), number of seeds on the main stem (0.4-0.8), total number of pods (0.2-0.6), total number of seeds (0.2- 0.7), 100 seeds weight (0.1-0.67), and moderate to low value for vegetative yield (0.1-0.5) and branching order (0.1-0.4). When looking at the data across locations (averaged data), the analysis of variance indicated that genotype (G) and environment (E) had a significant effect on all the traits analyzed (P< 0.001). The effect of genotype by environment interaction (GEI) was also highly significant (P< 0.001) for the majority of the traits but had no significant effect on vegetative yield. As shown in **Table 1**, heritability values across locations were higher for plant height (0.82) and flowering time (0.69), two traits well known for being under the influence of a high quantitative genetic component in many crops, as well as for the number of pods and seeds produced on the main stem (0.60-0.63). Contrarily, lower heritability values were estimated for the total number of pods and seeds produced (0.20-0.35), and almost null for the number of branching orders (0.06). Overall, variance component estimates showed a preponderant effect of the environment (**Table 1**). For this reason, the genetic analysis of these traits should be conducted on the singular specific environments separately.

**Table 1:** Estimates of heritability (H_2_) of breeding traits in single locations (from Gulisano et al., 2022a) and across trials.

	EC	PT	NL-Sc	NL-Wi	Across trials					
	*H_2_*	*H_2_*	*H_2_*	*H_2_*	G	E	Block	GEI	ε	H_2_
Plant height	0.72	0.86	0.56	0.57	16.4	45.1	1.9	3.8	32.9	0.82
Flowering time	–	0.93		0.87	10.8	86.6	0.2	1.4	0.9	0.88
Branching orders	0.33	0.44	0.46	0.07	0.4	44.5	2.7	0.4	42.9	0.06
Vegetative yield	–	0.54	0.17	0.21	3.5	27.1	5.0	1.5	62.9	0.39
Pods on main stem	0.48	0.42	0.83	0.46	6.2	57.0	0.8	6.6	29.5	0.60
Seeds on main stem	0.46	0.57	0.83	0.53	4.7	70.2	0.3	4.7	20.4	0.63
Pods Total	0.23	0.41	0.69	0.58	1.4	50.0	1.1	9.6	38.0	0.20
Seeds Total	0.21	0.36	0.70	0.64	2.3	58.2	0.9	6.1	32.5	0.35
100 Seed weight	0.67	0.17	0.63	0.24	2.7	39.5	0.2	3.5	54.1	0.33

For the analysis of variance across trials, the percentage of variance explained by the different components of variance is reported. Total Variance was decomposed in: genotype (G), environment (E), block effects within trial (Block), interaction of genotype by environment (GEI) and Residuals (ε) effects, reported as percentage of the total variance. Traits that were not measured are indicated with a dash. Crossed out traits were discarded for this study.

### 3.3 Genome wide association studies

The GWAS analysis was performed using the R package StatGenGWAS. The Kinship relationship matrix among samples, calculated with the Van Raden method, was included together with the country of origin of the accessions as covariate, to adjust for population structure. The quantile distribution of the observed p-values versus the expected p-values (QQ plot) confirmed adequacy of the population structure correction.

**Figure 2:**
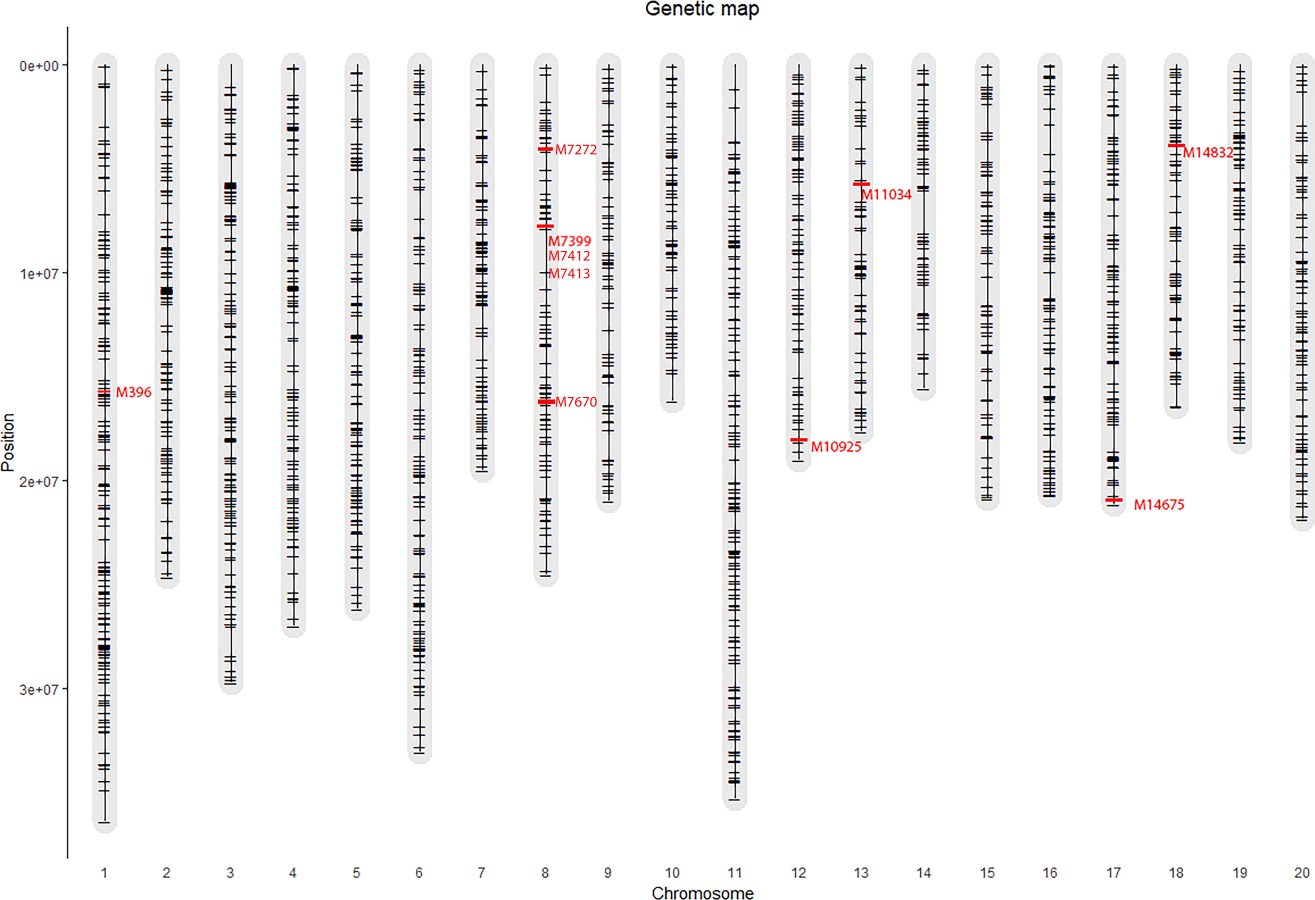
Markers distribution on the 20 chromosomes of *L. angustifolius* genome. In red, significant markers detected in association with agronomic traits using Bonferroni threshold (LOD 5.52, experiment *wise error α = 0.05)*.

**Figure 3:**
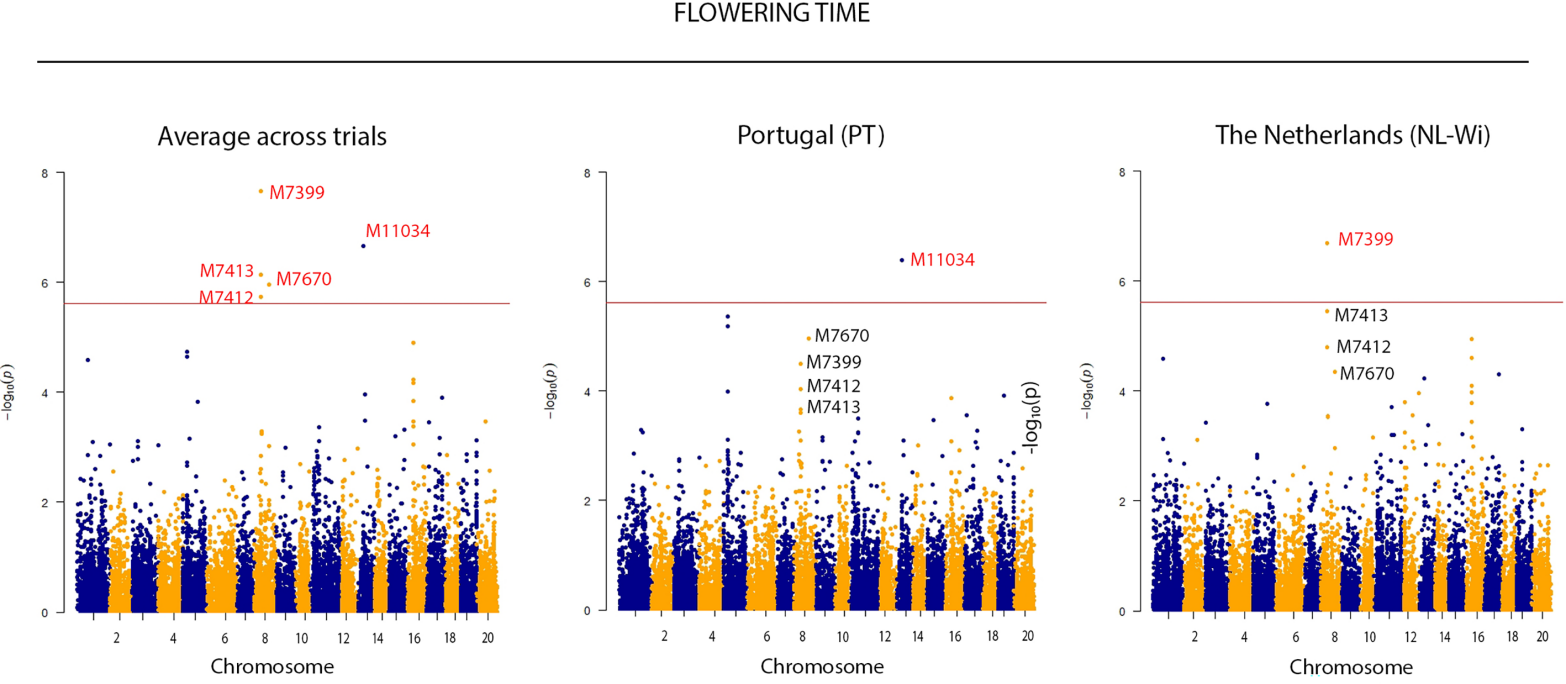
Manhattan plots displaying SNP markers-trait associations identified for Flowering time in GWAS using 223 accessions of *L. mutabilis*. The red line indicates the Bonferroni threshold (LOD = 5.52). Common SNPs showed significant association with variation in flowering time across locations, even if not all of them were detected as significant.

**Figure 4:**
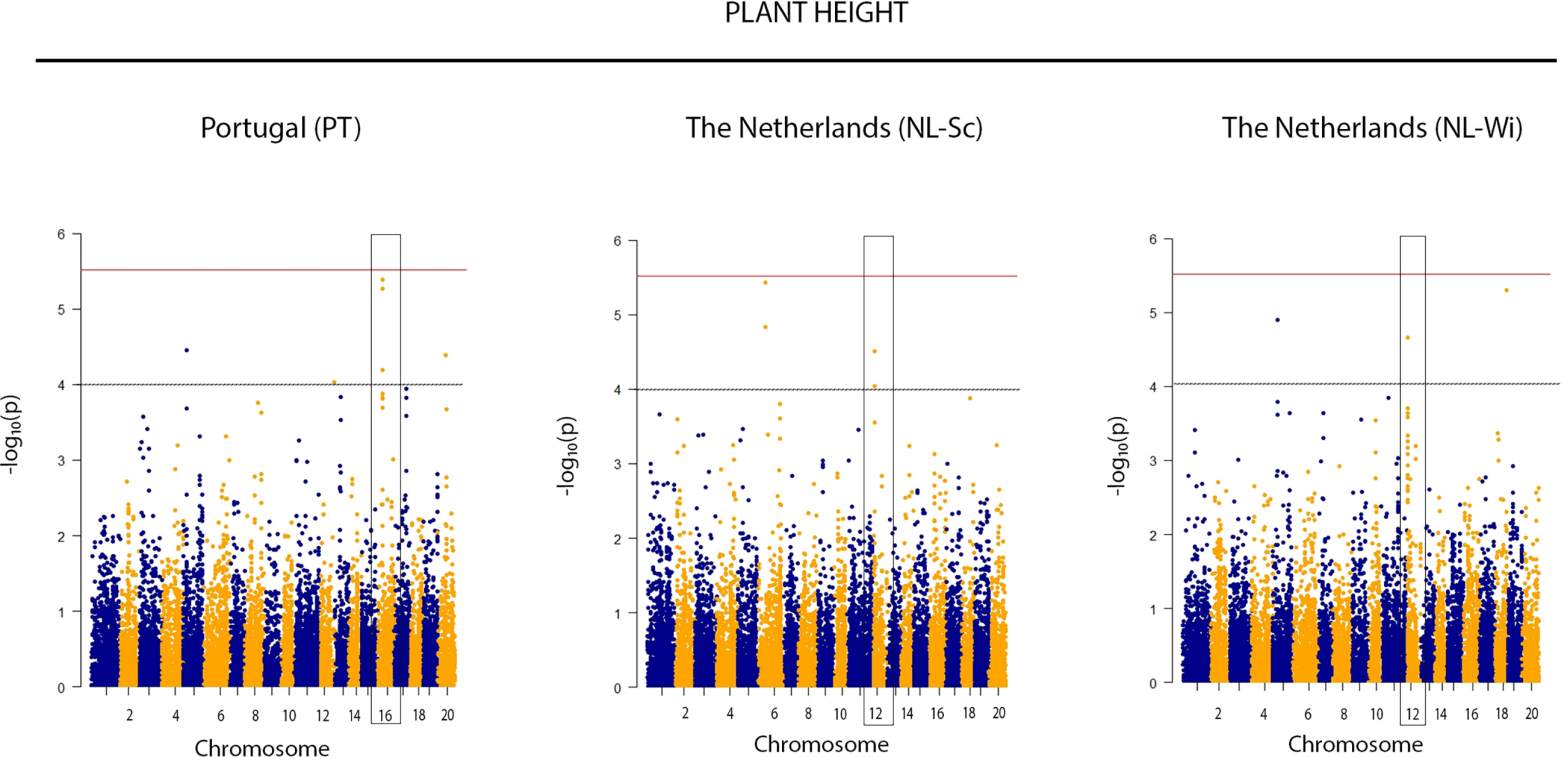
Manhattan plots suggestive of a SNP markers-trait association for plant height on chromosome LG16 and LG12, respectively for *L. mutabilis* growing in Mediterranean (Portugal) and North-Central European cropping conditions *(NL-Sc and NL-Wi).* The red line indicates the Bonferroni threshold (LOD = 5.52). The black dotted line indicates a suggestive threshold of LOD= 4. The rectangles highlight interesting piling up of SNPs below the threshold of significance, harboring interesting candidate genes related to plant height.

Due to the large effect of genotype by environment interaction on the phenotypic variation, GWAS was performed analyzing each trial separately, as well as across all the locations. By applying the Bonferroni threshold (–log_10_ (p) > 5.52), a total of 5 significant SNPs were identified as associated with flowering time, 1 SNP was associated with plant height, 1 SNP with vegetative yield and 4 SNPs with the number of pods on the main stem (Pods MS) (reported in **Figure 2** and **Table 2**). Contrarily, no significant SNPs were found in association with the remaining phenotypic traits. Single location GWAS for flowering time revealed the association of the SNP M11043, located on chromosome 13, with flowering time in the Portuguese environment. M11034 explained ~6% of the phenotypic variance observed. In NL-Wi, SNP M7399 on chromosome 8 was found to explain almost 14% of variation in time to flowering. When the two trials were analyzed together, both M11034 and M7399 remained significantly associated to flowering time explaining respectively 5.4 and 13.2% of variation across trials. Additionally, M7412, M7413 and M7670 on chromosome 8 were also detected as significant across trials, explaining between 7 and 14% of variation in flowering time. Notably, it was possible to observe a similar piling up of SNPs on chromosome 8 also in PT, harboring some of the same markers detected across trials in association with flowering time (M7670 and M7399), but below the stringent Bonferroni detection threshold (**Figure 3**). Instead, the SNP M11034 was also associated with the number of Pods MS in the trial located in the Netherlands in 2019 (NL-Sc). For Pods MS, one more SNP was detected on chromosome 8 across trials (M7272), while two more SNPs were detected on single trials respectively on chromosome 18 (M14832) in Ecuador and chromosome 17 (M14675) in NL-Sc. About 10% of the plant height variation in Ecuador was ascribable to the SNPs M396 on chromosome 1, while no other SNPs associated with this trait passed the Bonferroni threshold in the other locations, despite the high heritability and genetic variation recorded. However, the visual inspection of Manhattan plots revealed the presence of different regions likely associated with plant height in the other trials (**Figure 4**). The importance of these SNPs needs to be further investigated considering that Bonferroni is a quite conservative threshold. As an example, an interesting piling up of SNPs right below the threshold was observed on chromosome 16 for PT, while another piling up in association with this trait was observed on chromosome 12 for both NL-Sc and NL-Wi. Finally, 1 SNP associated to vegetative yield was detected on chromosome 12 (M10925), but only in NL-Sc. The SNPs detected as significant are reported in **Table 2**.

**Table 2:** SNPs found in significant association with plant-architecture and yield related traits for 223 accessions of *L. mutabilis* grown in four different environments.

Traits	Markers	Chr	Position	Environment	LOD	% Var	Effect	N. of genes
Flowering time (days)	**M11034**	LG13	5,615,305	PT	6.39	6.35	15.49	14
	**M7399**	LG08	7,668,768	NL-Wi	6.69	13.9	9.44	20
	**M11034**	LG13	5,615,305	All Trials	6.65	5.48	16.12	14
	**M7399**	LG08	7,668,768	All Trials	7.67	13.2	7.65	20
	M7412	LG08	7,670,152	All Trials	5.74	7.25	14.29	20
	M7413	LG08	7,680,541	All Trials	6.14	8.16	7.72	18
	M7670	LG08	16,132,804	All Trials	5.95	14.33	-21.86	1
Plant height (cm)	M396	LG01	15,690,000	EC	5.99	10.3	14.24	2
Vegetative yield (g)	M10925	LG12	18,150,269	NL-Sc	5.95	19.3	-168.78	29
Pods on the main stem (number)	**M11034**	LG13	5,615,305	NL-Sc	5.99	5.93	-5.67	14
	M14832	LG18	3,860,231	EC	5.62	10.7	9.02	5
	M14675	LG17	20,841,361	NL-Wi	6.03	7.00	4.03	16
	M7272	LG08	4,062,540	All Trials	5.65	4.75	12.41	5

SNPs that are detected in more than one environment, or in association with more than one trait, are reported in bold. For each marker we report: the position on *L. angustifolius* chromosome (Chr), the trial where the association was detected as significant (Environment), the LOD value of association, the phenotypic variance explained (% Var), the allelic effect on the phenotypic mean of the trait (Effect) and the number of genes found in a window of ± 80 kbp from the marker.

### 3.4 Putative candidate genes

We searched for putative candidate genes in regions close to the peak of the eight QTLs detected. An interval of ± 80 kbp around the peak of these QTLs was taken as search window for candidate genes as this is about the size of the linkage disequilibrium blocks, hence genes outside this window are not likely to be causative for the QTL effect. All search regions together harbored a total of 91 unique genes on the *L. angustifolius* genome. Moreover, the investigation of other interesting regions previously mentioned as likely associated with plant height, which did not pass the stringent Bonferroni correction, led to the identification of 37 additional genes, mostly related to plant development. The investigation of these genes, based on functional annotation of their orthologs on *Arabidopsis* genome and relevant literature, resulted in a subset of 10 genes as possibly involved in the control of traits of interest. These genes, listed in **Table 3**, are known to play a role in regulation of flowering and are involved in different plant growth and development processes. For instance, the identified genes encoding the protein FANTASTIC FOUR 3 FAF3, and the transcription factors LNK3 and GAMYB, are known to contribute to the genetic control of flowering time in Arabidopsis and other species (Wahl et al., 2010). *FAF3* is located on chromosome 8 at a distance of 77 kbp from the SNP M7399 associated with flowering, while *LNK3* and *GAMYB* are located on chromosome 13 (respectively, 62 kbp upstream and 44 kbp downstream of SNP M11034). A gene encoding an E3-ubiquitin protein ligase RING1 was detected on chromosome 17 in linkage with the SNPs M14675, that showed significant association with Pods MS. Members of the E3 ubiquitin ligases are well known to play a role in plant growth and development. Additionally, important genes involved in plant growth were identified on chromosome 12 and 16. Genetic polymorphism in these genes may be associated with plant height and vegetative yield and captured by blocks of closely located markers: M12928 to M12945 on chromosome 16 and M10488, M10515 and M10527 on chromosome 12 (**Figure 4**). These included genes encoding transcription factors involved in vascular development (*RF2b*), shade-induced plant growth (*HAT3*), transcriptional regulators of flowering time (*Dr1, BIM1*) and genes involved in the gibberellin signaling pathway (*GA3OX1*).

**Table 3:** Functional annotation of ortholog genes proposed as potential candidate genes for plant architecture and yield-related traits in *L. mutabilis*.

Trait	Chr	Candidate genes	Function annotation/common name
Plant height (PT)	LG16*	LOC109329023	transcription factor RF2b-like
Plant height (NL)	LG12*	LOC109362850	homeobox-leucine zipper protein HAT3
Vegetative yield (NL-Sc)	LG12	LOC109361654	gibberellin 3-beta-dioxygenase 1/GA3OX1
	LG12	LOC109362161	histone deacetylase 15/HDA15
	LG12	LOC109362682	transcription factor BIM1
	LG12	LOC109362691	protein Dr1 homolog
Flowering	LG08	LOC109354086	protein FANTASTIC FOUR 3
Flowering (PT)/ Pods main stem (NL-Sc)	LG13	LOC109325309	protein LNK3
	LG13	LOC109363186	transcription factor GAMYB
Pods main stem (NL-Wi)	LG17	LOC109330763	E3 ubiquitin-protein ligase RING1

*Putative candidate genes found in linkage disequilibrium with SNPs detected below the Bonferroni threshold (LOD ≥ 4).

## 4 Discussion

Large phenotypic variation for plant height, flowering time, biomass and yield related traits was observed among the 223 *L. mutabilis* accessions studied, indicating that the used panel is suitable for a GWAS study that requires genetic variation of the traits. The large phenotypic variation observed in our population is beneficial to select breeding candidates in order to initiate a genetic improvement program aimed at developing superior varieties adapted to Europe and high performing. One of the key factors to promote the cultivation of high yielding *L. mutabilis* in Europe is breeding towards earliness and semi-determinate growth habit (Caligari et al., 2000; Gulisano et al., 2022a). In our extensive phenotypic evaluation of this collection, we highlighted the presence of strong correlations between plant height, flowering time and seed yield (Gulisano et al., 2022a). These findings are in line with previous observations carried out in other legumes (González et al., 2016; Annicchiarico et al., 2021). Breeding for plant architecture should be tailored to specific growing conditions, considering that environmental conditions highly impact the morphology and yield of *L. mutabilis* (Gulisano et al., 2022a). Generally, restricted plant architecture, caused by a limited number of branching orders, is associated with higher seed yield in *L. mutabilis*. In fact, indeterminate growth and extensive branching have a detrimental effect on seed yield, since Andean lupin tends to produce the largest share of seeds on the main stem and on the first branching order (Sinjushin, 2015; Gulisano et al., 2022a). However, from our study emerges that total seed production positively correlates with late flowering and higher vegetative biomass when Andean Lupin is cultivated in Mediterranean cropping conditions (Portugal). This observation supports the hypothesis that plants with indeterminate growth, thus larger branching orders, outperform determinate varieties in adverse climates. One possible explanation is that, under high temperature or drought, the indeterminate growth habit may better compensates yield loss, which is more evident in determinate types, where pods set is stronger negatively affected by the environmental factors (Martins et al., 2016; Gulisano et al., 2022a). However, it is important to report that extensive branching in Mediterranean conditions is already constrained by environmental factors such as water scarcity, hence even plants characterized by an indeterminate growth habit will not produce more than two branching orders, maintaining overall a semi-determinate growth. Yet, the effect that a larger contribution of higher branching orders to seed yield can have on seed quality and composition remains to be further investigated.

Given the importance of breeding for earliness and growth habit in this crop, the collection under study showed enough phenotypic and genetic diversity in flowering time (about one month difference between early and late flowering genotypes), and vegetative yield, to potentially breed for novel early and high yielding lines. The highlighted variation is therefore suitable to support breeding aimed at the introduction of *L. mutabilis* as a protein and oil crop in Europe. For genetic improvement purposes, understanding the genetic architecture of earliness, plants architecture and seed-yield related traits is pivotal. By evaluating strategic breeding parameters including heritability and genotype-by-environment interactions (GxE), we demonstrated that some phenotypic traits of interest are highly influenced by their genetic components. We report high heritability values for flowering (H^2^ = 0.88), plant height (H^2^ = 0.82) and production of pods and seeds on the main stem (H^2^ = 0.6) across the different trials, thus across environments and growing conditions (**Table 1**). Our results are in line with the heritability values reported for plant height, productivity on the main stem and flowering time by previous authors (Hardy et al., 1998; Guilengue et al., 2020). These findings encourage breeding towards early varieties with restricted plant architecture and improved yield in Europe, since high heritability is always advantageous to increase the expected genetic gain in crop selection.

To the best of our knowledge, this is the first genome-wide association study (GWAS) investigating the genetic architecture of agronomic traits in a collection of *L. mutabilis*. Since genotype by environment interaction can alter the QTLs effects, an initial GWAS analysis carried out on single trial data, followed by a genome-wide scan across all locations, was considered the best approach to analyze our data. We have already shown in previous studies that in the absence of a reference genome for *L. mutabilis*, the use of *L. angustifolius* pseudochromosomes assembly can be suitable for genome-wide associations in this species (Gulisano et al., 2022b). In addition, our GWAS approach also shows that, in the absence of pure highly stable homozygous lines, a mapping experiment can be conducted using allele frequencies as marker score, estimated at each marker locus in pooled DNA samples. We estimated allele frequencies by counting the number of reads carrying the reference alleles on the total number of generated reads at a given variant position. In order to ensure a more accurate estimation of allele frequencies, as well as accounting for the presence of alleles of different individuals in the same DNA sample, we have discarded SNPs with a reads depth<45x from the final markers set. This approach of treating markers as a variable with a continuous distribution was already adopted by Petit et al. (2020), and is a good option for mapping studies in crops where a certain degree of cross-pollination is expected within the accessions. The Efficient Mixed Model Association (EMMA) approach showed robust performance on the traits and in the population studied. In GWAS, false discoveries are a major concern and spurious associations between phenotypes and testing markers can arise as a consequence of population structure and differences in relatedness among individuals in the tested population (Sul et al., 2018). The association model we used incorporated kinship and the origin of accessions as covariates to control for spurious association and false discoveries. The QQ-plots generated after the genome scan confirmed the high quality of the GWAS models. The difference between observed and expected −log10(p) values, in fact, showed no inflation and values of inflation factors close to the ideal value of 1 for all the traits.

GWAS analysis identified a total of 5 significative SNPs associated with flowering time (M11034, chr 13, position 5,615,305; M7399, chr 8, position 7,668,768; M7412 chr 8, position 7,670,152; M7413, chr 8, position 7,680,541; M7670, chr 8, position 16,132,804), 1 SNP associated with plant height (M369, chr 1, position 15,690,000), 1 SNP associated with vegetative yield (M10925, chr 12, position 18,150,269), and 3 SNPs associated with number of pods on the main stem (M1134, chr 13, position 5,615,305; M14832 on chr 18, position 3,860,231; M14675, chr 17, position 20,841,361). In agreement with the idea that the genetic control of agronomic traits might differ in response to different environmental conditions, our study points out that the association of genomic regions with phenotypic traits can be location specific, thus different QTLs can be detected in different environments. Indeed, most of the SNPs reported were identified as single location markers. Taking into account that the Bonferroni correction method is a very conservative statistics to set a threshold *P-*value (Kaler and Purcell, 2019), we have also investigated some genomic regions where SNPs were piling up in the QQ plots, even if they were not passing the stringent Bonferroni threshold. We therefore report on likely association on chromosome 12 and chromosome 16 for plant height in the Netherlands (NL-Sc and NL-Wi) and in Portugal (PT), respectively.

Flowering time is a quantitative traits and its genetic control has been investigated in several crops, including *L. albus*, where it has been described as quantitative and under the control of major QTLs and numerous regulatory genes, including *FLOWERING LOCUS T (Fta1), CONSTANS (CO), FY, MOTHER OF FT AND TERMINAL FLOWERING LOCUS 1 (TFL1)*, but also of genes related to the response to photoperiod and vernalization, as well as regions involved in hormone signaling pathways (i.e, gibberellin) (Rychel et al., 2019). In our panel, we did not detect these major candidate genes conserved in several crops. However, the variation in flowering time in our panel resulted associated with two large QTL on chromosome 8 (one identified by the SNPs M7399, M7412 and M7413 and one indicated by the SNP M7670) with a QTL on chromosome 13 (M11043). In addition, single trials analysis revealed that the effect of the QTL on chromosome 8 is more relevant in NL, while the effect of the QTL on chromosome 13 is more relevant in the Mediterranean environment. Given that M7399 and M7670 respectively accounted for ~11% and 16% of the phenotypic variation observed across trials, it is possible to hypothesize the presence of major QTLs in their surrounding genomic regions. Our investigation highlighted the presence of the gene *FANTASTIC FOUR 3* (*FAF3*) at a distance between 65 kb and 77 kb from M7413, M7412 and M7399, thus located in a linkage disequilibrium block captured by these three proximal markers. *FAF3* is a member of the FANTASTIC FOUR (FAF) genes family, which contains four members in *Arabidopsis* (*FAF1*-*FAF4*), dynamically expressed throughout development (Wahl et al., 2010), and mainly involved in the modulation of the shoot meristem size. Wahl et al., 2010 reported *FAF* genes as redundant in function, indicating that all the four proteins may perform a very similar role. Interestingly, the *FAF* genes are expressed in flowering buds and inflorescence, with an increased expression during the transition to flowering. Moreover, a regulatory interaction has been reported between *FAF2* and *FAF4* with the WUSCHEL (WU)-CLAVATA (CLV3) signaling pathway that plays a central role in regulating stem cell proliferation and differentiation in crop plants, supporting organogenesis in the floral meristem through a complex molecular pathway (Wahl et al., 2010). Notably, studies on *Arabidopsis thaliana* report *FAF1/FAF2* and *FAF3/FAF4* to be recently duplicated paralogs (Blanc and Wolfe, 2004) that could therefore be characterized by similar structure and functions. Unfortunately, it was not possible to detect any annotated and characterized gene in the genomic region surrounding M7670, hence further studies are needed to elucidate which gene is responsible for the variation in flowering time associated with this SNP. In addition to these markers detected on chromosome 8, we report the finding of SNP M11043 on chromosome 13 as associated to flowering time across location and in Mediterranean growing conditions specifically. M11043 is located in a genomic area that harbors two genes potentially involved in the regulation of flowering. The gene *GAMYB* is located at a distance of 44.2 kbp from the detected marker, while the gene *NIGHTLIGHT–INDUCIBLE AND CLOCK-REGULATED 3 (LNK3)* at a distance of 62.3 kbp. *GAMYB* is described as a regulator of flower induction, *via* the transcriptional activation of the LEAFY (*LFY*) gene (Gocal et al., 1999; Zhang et al., 2020), that results in early flowering both in dicots and monocot (Weigel and Nilsson, 1995; He et al., 2000). Instead, members of the family of *NIGHTLIGHT–INDUCIBLE AND CLOCK-REGULATED* (*LNK1* and *LNK2*) genes are known to control photomorphogenic and photoperiodic responses, as well as circadian rhythms (Rugnone et al., 2013). Recently, Li et al., 2021 obtained an early flowering line in soybean by targeted mutagenesis of the four *LNK2* genes, that molecularly interact with major flowering genes, showing that targeted breeding on these genes can contribute to soybean expansion to high latitudes in Europe where shorter growing cycles are needed (Li et al., 2021). Despite we report on *LNK3*, that was not previously described as involved in flowering, it will be beneficial to further investigate its role in *L. mutabilis*, based on the strong significant association detected, and the fact that the polymorphism detected explains ~6% of the flowering variation recorded.

The genetic analysis carried out in this study showed that plant height and vegetative yield are likely affected by QTL-by-environment interaction, since the detected SNPs and the respective candidate QTLs in linkage with them appeared to be environment-specific. Hence, different transcriptional regulators can similarly affect the same traits in response to different environmental stimuli. In particular, plant height showed association with the SNP M396 (chr 1, position 15,690,000) in Ecuador, but a piling up of SNP (not significant based on Bonferroni) was instead observed on chromosome 16 in Portugal, and on chromosome 12 in the Netherlands. On chromosome 1 we did not report any relevant candidate genes linked to M396 and plant height. Contrarily, we report the finding of a transcription factor *RF2b-like* and a homeobox-leucine zipper protein *HAT3*, both harbored in the genomic regions where we visualize an interesting piling up of SNPs, that likely resulted as false negative when applying Bonferroni threshold to detect significant associations. These genes are respectively involved in vascular development and shoot tissue organization (*RF2b)* and in shade-induced plant growth (*HAT3)*, and can both lead, through different mechanisms, to the development of semi-dwarf varieties (Dai et al., 2004; Bou-Torrent et al., 2012).

The association between genetic variants and vegetative yield was only significant in North-Central European summer conditions, where the SNP M10925 (chromosome 12, position 18,150,269) was detected. M10925 showed a strong association, and it is able to explain a large share (~20%) of the phenotypic variation. This suggest the presence of a major QTL, considering that vegetative yield is a quantitative and complex trait. Harbored in this area, we identified 4 different candidate genes: *GIBERELLIN 3-BETA-DIOXYGENASE1 (GA30X1)*, *HISTONE-DEACETYLASE15 (HDA15)*, transcription factor *BIM1* and *D*r1 homolog, which are gene involved in the gibberellin pathway (*GA30X1*) and regulation of flowering time (*BIM, Dr1*). On one hand, *GA30X1* is involved in the gibberellin pathways as the enzyme catalyzing the final step of the synthesis of bioactive gibberellin during vegetative growth, and its negative regulation might directly lead to substantial decreases in biomass production and to the development of semidwarf types (Hu et al., 2008). On the other hand, transcriptional regulators of flowering time can promote flowering in response to hormonal shifts or environmental stimuli and, as a consequence of the strong interplay between flowering time and growth habit in legumes, indirectly act on biomass production and vegetative yield. For example, the transcription factor *BIM1*, part of the bHLH family of transcription factors, can lead to the inhibition of floral transition in Arabidopsis by transduction of hormonal brassinosteroid signals to the activation of *FLOWERING LOCUS C* (*FLC*) and consequent floral repression, promoting in turn vegetative growth. Conversely, defects in *BIM1* can lead to early floral transition (Li et al., 2018). Instead, *Dr1* and *HDA15* play important roles in determining plant response to different environmental stimuli, such as drought and elevated ambient temperature (Zotova et al., 2019; Shen et al., 2019). For example, it is suggested that Dr1 protein can bind and deactivate genes sensing environmental cues for drought, releasing in turn the activity of vernalization and flowering regulators (*Vrn1* ad *FT1*). This mechanism has been described as a successful drought escape strategy in wheat (Zotova et al., 2019), and could be linked in *L. mutabilis* to other successful escape strategies like accelerated seed maturation and pod filling, which rely on a preferential partitioning of nutrients towards reproductive growth, at the expense of vegetative growth.

Finally, four QTLs were also detected in association with number of pods produced on the main stem (Pods MS). One SNP was detected across trials (M7272, chr 8, position 4,062,540), while the remaining three SNPs indicated QTLs location-specific. M11034 (chromosome 13, position 5,615,305) was detected in the NL-Sc, M1483 (chromosome 18, position 3,860,231) in EC and M14675 (chromosome 17, position 20,841,361) in NL-Wi. On chromosome 17, we highlighted the presence of the E3 ubiquitin ligases *RING1* gene in linkage disequilibrium with the respective significant marker. E3 ubiquitin ligases have been documented to play an important role in the regulation of plant growth and development, such as seed dormancy and germination, root growth and flowering time control, as well as regulation of several abiotic stress responses (Zhang et al., 2015; Shu and Wenyu, 2017). Increasing numbers of studies have documented the key role of the different types of E3s, including E3-RING as involved in seed biology, root elongation, flowering time control, light response, ABA signaling transduction and response to drought and salinity stresses.

*L. mutabilis* breeding in Europe can beneficiate from the findings of this study as alleles for the QTLs can be typed and potentially used to assist the genetic selection. Furthermore, the highlighted association of genomic regions with the reported phenotypic traits represents a molecular framework for further deeper investigations of the intricate networks regulating plant development and phenology in this species.

## Data availability statement

The genotypic data used for the genome wide association study and generated by genotyping by restriction site-associated DNA sequencing (RAD-seq) are openly available at the 4TU. Research Data repository (http://doi.org/10.4121/21334146.v1).

## Author contributions

AG and LT conceived and designed the experiment. AG performed all the analysis, interpreted the data and wrote the manuscript. AL contributed to the interpretation of the data and the writing of the manuscript. LT wrote the proposal and revised the manuscript. M-JP and EL provided guidance in the statistical analysis and genome-wide association study and revised the manuscript. All authors read and approved the manuscript.
